# Supplementary material for: Microstructural asymmetries of the planum temporale predict functional lateralization of auditory-language processing
Source: eLife. 2024 Dec 16;13:RP95547. doi: 10.7554/eLife.95547 (PMC11649236; doi:10.7554/eLife.95547)
Supplement: Supplementary file 1. — (a) Group-level hemispheric asymmetry of planum temporale (PT) functional and structural measures. (b) The interaction effect of ‘PT structural AI × Heschl's gyrus (HG) gyrification pattern’ on PT nonspeech-related functional asymmetry indexes (AIs) (the first specificity analysis). (c) The correlations of PT structural AIs with PT nonspeech-related functional activation AIs after controlling for the HG gyrification pattern (the first specificity analysis). (d) The interaction effect of ‘PT structural measure × HG gyrification pattern’ on PT nonspeech-related functional activation for each hemisphere (the first specificity analysis). (e) The correlations of PT structural measures with PT nonspeech-related functional activation for each hemisphere after controlling for the HG gyrification pattern (the first specificity analysis). (f) The interaction effect of ‘PT structural AI ×HG gyrification pattern’ on speech-related functional AIs of the entire hemisphere (the second specificity analysis). (g) The correlations of PT structural AIs with speech-related functional AI of the entire hemisphere after controlling for the HG gyrification pattern (the second specificity analysis). (h) The interaction effect of ‘PT structural measure × HG gyrification pattern’ on speech-related functional activation of the entire ipsilateral hemisphere (the second specificity analysis). (i) The correlations of PT structural measures with speech-related functional activation of the entire ipsilateral hemisphere after controlling for the HG gyrification pattern (the second specificity analysis). (j) The difference in PT functional and structural metrics between groups with single and duplicated HG within each hemisphere (the effect size, Cohen’s D). [file elife-95547-supp1.docx]

Supplementary Materials1 for

**Microstructural asymmetries of the planum temporale predict functional lateralization of auditory-language processing**

Peipei Qin,^1, ⁋^ Qiuhui Bi,^2, 1, ⁋^ Zeya Guo,^1^ Liyuan Yang,^1^ Haokun Li,^1^ Peng Li,^1^ Xinyu Liang,^1^ Junhao Luo,^1^ Xiangyu Kong,^1^ Yirong Xiong,^1^ Bo Sun,^2^ Sebastian Ocklenburg,^3, 4, 5^ Gaolang Gong ^1, 6, 7, *^

*Corresponding author. gaolang.gong@bnu.edu.cn

**This file includes:**

supplementary file 1a-1j

Supplementary file 1a. Group-level hemispheric asymmetry of PT functional and structural measures

|  | **L1/R1** | | | **L1/R2** | | | | **L2/R1** | | | **L2/R2** | | | |
| --- | --- | --- | --- | --- | --- | --- | --- | --- | --- | --- | --- | --- | --- | --- |
|  | *T* | *P_FWE_* | *Cohen’s d* | | *T* | *P_FWE_* | *Cohen’s d* | *T* | *P_FWE_* | *Cohen’s d* | | *T* | *P_FWE_* | *Cohen’s d* |
| **Speech perception** | **6.82** | **<.001^*^** | **0.35** | | **5.55** | **<.001^*^** | **0.52** | -1.29 | >.99 | -0.10 | | 1.40 | >.99 | 0.26 |
| **Speech comprehension** | **6.54** | **<.001^*^** | **0.36** | | **6.12** | **<.001^*^** | **0.57** | -0.08 | >.99 | -0.12 | | 2.94 | >.99 | 0.12 |
| **Surface area** | **6.83** | **<.001^*^** | **0.42** | | **14.4** | **<.001^*^** | **1.48** | -2.06 | >.99 | **-**0.25 | | **4.88** | **<.001^*^** | **0.73** |
| **Thickness** | **-10.0** | **<.001^*^** | **-0.53** | | -3.08 | 0.06 | -0.30 | **-4.39** | **<.001^*^** | **-0.44** | | -3.58 | 0.01 | -0.49 |
| **Myelin content** | -0.99 | >.99 | -0.05 | | **5.97** | **<.001^*^** | **0.58** | **-6.50** | **<.001^*^** | **-0.68** | | 2.67 | 0.23 | 0.36 |
| **NDI** | **-4.43** | **<.001^*^** | **-0.21** | | **4.26** | **<.001^*^** | **0.40** | **-4.83** | **<.001^*^** | **-0.48** | | 2.59 | 0.29 | 0.30 |
| **ODI** | **26.7** | **<.001^*^** | **1.65** | | **15.8** | **<.001^*^** | **1.71** | **8.69** | **<.001^*^** | **1.03** | | **9.17** | **<.001^*^** | **1.49** |

*P_FWE_*, corrected *P* value after Bonferroni correction; *, significant hemispheric asymmetry between the left and right PTs; L1/R1, single HG on the left and single HG on the right; L1/R2, single HG on the left and duplicated HG on the right; L2/R1, duplicated HG on the left and single HG on the right; L2/R2, duplicated HG on the left and duplicated HG on the right; NDI, neurite density index; ODI, orientation dispersion index.

Supplementary file 1b. The interaction effect of “PT structural AI × HG gyrification pattern” on PT nonspeech-related functional AIs (the first specificity analysis)

|  | **Working memory** | | **Incentive processing** | | **Emotion processing** | | **Social cognition** | | **Motor** | | **Relational processing** | |
| --- | --- | --- | --- | --- | --- | --- | --- | --- | --- | --- | --- | --- |
|  | *F* | *P_FWE_* | *F* | *P_FWE_* | *F* | *P_FWE_* | *F* | *P_FWE_* | *F* | *P_FWE_* | *F* | *P_FWE_* |
| **Surface area** | 6.54 | 0.32 | 0.68 | >.99 | 3.94 | >.99 | 0.13 | >.99 | 0.20 | >.99 | 0.04 | >.99 |
| **Thickness** | 2.06 | >.99 | 0.00 | >.99 | 0.45 | >.99 | 1.06 | >.99 | 0.80 | >.99 | 0.20 | >.99 |
| **Myelin content** | 0.03 | >.99 | 2.86 | >.99 | 0.69 | >.99 | 0.01 | >.99 | 0.19 | >.99 | 0.53 | >.99 |
| **NDI** | 1.33 | >.99 | 0.84 | >.99 | 0.01 | >.99 | 0.28 | >.99 | 0.03 | >.99 | 0.19 | >.99 |
| **ODI** | 0.32 | >.99 | 0.05 | >.99 | 0.02 | >.99 | 2.24 | >.99 | 0.35 | >.99 | 3.71 | >.99 |

PT, planum temporale; HG, Heschl's gyrus; AI, Asymmetry index; *P_FWE_*, corrected *P* value after Bonferroni correction; NDI, neurite density index; ODI, orientation dispersion index.

Supplementary file 1c. The correlations of PT structural AIs with PT nonspeech-related functional activation AIs after controlling for the HG gyrification pattern (the first specificity analysis)

|  | **Working memory** | | **Incentive processing** | | **Emotion processing** | | **Social cognition** | | **Motor** | | **Relational processing** | |
| --- | --- | --- | --- | --- | --- | --- | --- | --- | --- | --- | --- | --- |
|  | *R* | *P_FWE_* | *R* | *P_FWE_* | *R* | *P_FWE_* | *R* | *P_FWE_* | *R* | *P_FWE_* | *R* | *P_FWE_* |
| **Surface area** | **0.18** | **<.001^*^** | 0.09 | 0.25 | **0.21** | **<.001^*^** | 0.05 | >.99 | **0.18** | **<.001^*^** | **0.14** | **<.01^*^** |
| **Thickness** | 0.03 | >.99 | 0.04 | >.99 | 0.09 | 0.17 | -0.01 | >.99 | -0.08 | 0.55 | -0.01 | >.99 |
| **Myelin content** | 0.01 | >.99 | 0.02 | >.99 | 0.06 | >.99 | 0.04 | >.99 | **-0.11** | **0.04^*^** | -0.06 | >.99 |
| **NDI** | 0.08 | 0.64 | -0.02 | >.99 | 0.09 | 0.26 | 0.01 | >.99 | -0.08 | 0.79 | -0.06 | >.99 |
| **ODI** | -0.03 | >.99 | 0.02 | >.99 | -0.02 | >.99 | 0.08 | 0.71 | -0.08 | 0.66 | 0.00 | >.99 |

PT, planum temporale; HG, Heschl's gyrus; AI, Asymmetry index; *P_FWE_*, corrected *P* value after Bonferroni correction; NDI, neurite density index; ODI, orientation dispersion index. *, significant correlation between PT functional and structural AIs

Supplementary file 1d. The interaction effect of “PT structural measure × HG gyrification pattern” on PT nonspeech-related functional activation for each hemisphere (the first specificity analysis)

|  |  | **Working memory** | | **Incentive processing** | | **Emotion processing** | | **Social cognition** | | **Motor** | | **Relational processing** | |
| --- | --- | --- | --- | --- | --- | --- | --- | --- | --- | --- | --- | --- | --- |
|  |  | *F* | *P_FWE_* | *F* | *P_FWE_* | *F* | *P_FWE_* | *F* | *P_FWE_* | *F* | *P_FWE_* | *F* | *P_FWE_* |
| **Left** | **Surface area** | 0.31 | >.99 | 0.81 | >.99 | 4.96 | >.99 | 1.93 | >.99 | 6.30 | 0.74 | 0.00 | >.99 |
|  | **Thickness** | 0.17 | >.99 | 0.93 | >.99 | 0.20 | >.99 | 0.56 | >.99 | 4.59 | >.99 | 1.04 | >.99 |
|  | **Myelin content** | 2.03 | >.99 | 2.24 | >.99 | 0.00 | >.99 | 7.06 | 0.48 | 4.23 | >.99 | 0.34 | >.99 |
|  | **NDI** | 0.79 | >.99 | 0.59 | >.99 | 0.11 | >.99 | 6.02 | 0.86 | 0.02 | >.99 | 0.18 | >.99 |
|  | **ODI** | 1.27 | >.99 | 0.01 | >.99 | 0.00 | >.99 | 0.15 | >.99 | 4.06 | >.99 | 0.31 | >.99 |
| **Right** | **Surface area** | 0.03 | >.99 | 0.03 | >.99 | 0.31 | >.99 | 0.24 | >.99 | 5.97 | 0.89 | 0.03 | >.99 |
|  | **Thickness** | 0.73 | >.99 | 1.13 | >.99 | 0.90 | >.99 | 0.03 | >.99 | 1.83 | >.99 | 5.24 | >.99 |
|  | **Myelin content** | 0.72 | >.99 | 1.14 | >.99 | 4.44 | >.99 | 1.10 | >.99 | **11.93** | **0.03^*^** | 1.68 | >.99 |
|  | **NDI** | 0.06 | >.99 | 0.12 | >.99 | 1.37 | >.99 | 4.59 | >.99 | 2.86 | >.99 | 1.03 | >.99 |
|  | **ODI** | 0.02 | >.99 | 1.87 | >.99 | 2.53 | >.99 | 2.00 | >.99 | 4.21 | >.99 | 0.18 | >.99 |

PT, planum temporale; HG, Heschl's gyrus; *P_FWE_*, corrected *P* value after Bonferroni correction; NDI, neurite density index; ODI, orientation dispersion index. *, the gyrification pattern of HG showed no significant effect on the correlation between functional and structural measures.

Supplementary file 1e. The correlations of PT structural measures with PT nonspeech-related functional activation for each hemisphere after controlling for the HG gyrification pattern (the first specificity analysis)

|  |  | **Working memory** | | **Incentive processing** | | **Emotion processing** | | **Social cognition** | | **Motor** | | **Relational processing** | |
| --- | --- | --- | --- | --- | --- | --- | --- | --- | --- | --- | --- | --- | --- |
|  |  | *R* | *P_FWE_* | *R* | *P_FWE_* | *R* | *P_FWE_* | *R* | *P_FWE_* | *R* | *P_FWE_* | *R* | *P_FWE_* |
| **Left** | **Surface area** | -0.02 | >.99 | -0.09 | 0.42 | 0.00 | >.99 | 0.01 | >.99 | 0.01 | >.99 | -0.02 | >.99 |
|  | **Thickness** | -0.03 | >.99 | 0.04 | >.99 | 0.04 | >.99 | 0.04 | >.99 | 0.02 | >.99 | -0.06 | >.99 |
|  | **Myelin content** | -0.04 | >.99 | 0.02 | >.99 | -0.03 | >.99 | 0.03 | >.99 | -0.05 | >.99 | -0.04 | >.99 |
|  | **NDI** | 0.06 | >.99 | -0.05 | >.99 | 0.00 | >.99 | -0.03 | >.99 | -0.04 | >.99 | -0.03 | >.99 |
|  | **ODI** | -0.03 | >.99 | -0.05 | >.99 | -0.10 | 0.42 | 0.03 | >.99 | -0.04 | >.99 | 0.02 | >.99 |
| **Right** | **Surface area** | -0.05 | >.99 | -0.03 | >.99 | -0.05 | >.99 | **-0.12** | **0.02^*^** | 0.06 | >.99 | -0.06 | >.99 |
|  | **Thickness** | 0.05 | >.99 | 0.01 | >.99 | 0.08 | 0.75 | -0.05 | >.99 | -0.01 | >.99 | 0.00 | >.99 |
|  | **Myelin content** | -0.05 | >.99 | 0.01 | >.99 | 0.02 | >.99 | -0.01 | >.99 | **-0.23** | **<.001^*^** | -0.09 | 0.43 |
|  | **NDI** | 0.00 | >.99 | 0.01 | >.99 | -0.01 | >.99 | 0.00 | >.99 | **-0.14** | **<.01^*^** | -0.11 | 0.15 |
|  | **ODI** | -0.02 | >.99 | 0.03 | >.99 | 0.06 | >.99 | 0.01 | >.99 | **-0.25** | **<.001^*^** | 0.00 | >.99 |

PT, planum temporale; HG, Heschl's gyrus; *P_FWE_*, corrected *P* value after Bonferroni correction; NDI, neurite density index; ODI, orientation dispersion index. *, significant correlation between PT functional activation and ipsilateral structural measures.

Supplementary file 1f. The interaction effect of “PT structural AI × HG gyrification pattern” on speech-related functional AIs of the entire hemisphere (the second specificity analysis)

|  | | **Entire hemisphere** | | | |
| --- | --- | --- | --- | --- | --- |
|  |  | **Speech perception** | | **Speech comprehension** | |
|  |  | *F* | *P_FWE_* | *F* | *P_FWE_* |
| **PT** | **Surface area** | 0.10 | >.99 | 0.27 | >.99 |
|  | **Thickness** | 0.15 | >.99 | 0.02 | >.99 |
|  | **Myelin content** | 0.10 | >.99 | 2.24 | >.99 |
|  | **NDI** | 0.05 | >.99 | 0.59 | >.99 |
|  | **ODI** | 0.38 | >.99 | 1.34 | >.99 |

PT, planum temporale; HG, Heschl's gyrus; AI, Asymmetry index; *P_FWE_*, corrected *P* value after Bonferroni correction; NDI, neurite density index; ODI, orientation dispersion index.

Supplementary file 1g. The correlations of PT structural AIs with speech-related functional AI of the entire hemisphere after controlling for the HG gyrification pattern (the second specificity analysis)

|  | | **Entire hemisphere** | | | |
| --- | --- | --- | --- | --- | --- |
|  |  | **Speech perception** | | **Speech comprehension** | |
|  |  | *R* | *P_FWE_* | *R* | *P_FWE_* |
| **PT** | **Surface area** | -0.05 | >.99 | 0.03 | >.99 |
|  | **Thickness** | -0.07 | 0.47 | -0.05 | >.99 |
|  | **Myelin content** | -0.02 | >.99 | 0.06 | 0.89 |
|  | **NDI** | -0.06 | >.99 | 0.05 | >.99 |
|  | **ODI** | 0.02 | >.99 | 0.02 | >.99 |

PT, planum temporale; HG, Heschl's gyrus; AI, Asymmetry index; *P_FWE_*, corrected *P* value after Bonferroni correction; NDI, neurite density index; ODI, orientation dispersion index.

Supplementary file 1h. The interaction effect of “PT structural measure × HG gyrification pattern” on speech-related functional activation of the entire ipsilateral hemisphere (the second specificity analysis)

|  | | **Entire ipsilateral hemisphere** | | | |
| --- | --- | --- | --- | --- | --- |
|  |  | **Speech perception** | | **Speech comprehension** | |
|  |  | *F* | *P_FWE_* | *F* | *P_FWE_* |
| **Left PT** | **Surface area** | 3.74 | >.99 | 0.63 | >.99 |
|  | **Thickness** | 0.11 | >.99 | 0.86 | >.99 |
|  | **Myelin content** | 0.31 | >.99 | 0.86 | >.99 |
|  | **NDI** | 0.39 | >.99 | 1.25 | >.99 |
|  | **ODI** | 0.15 | >.99 | 1.35 | >.99 |
| **Right PT** | **Surface area** | 0.44 | >.99 | 0.30 | >.99 |
|  | **Thickness** | 0.45 | >.99 | 1.35 | >.99 |
|  | **Myelin content** | 0.52 | >.99 | 0.10 | >.99 |
|  | **NDI** | 0.71 | >.99 | 0.03 | >.99 |
|  | **ODI** | 3.68 | >.99 | 0.00 | >.99 |

PT, planum temporale; HG, Heschl's gyrus; *P_FWE_*, corrected *P* value after Bonferroni correction; NDI, neurite density index; ODI, orientation dispersion index.

Supplementary file 1i. The correlations of PT structural measures with speech-related functional activation of the entire ipsilateral hemisphere after controlling for the HG gyrification pattern (the second specificity analysis)

|  | | **Entire ipsilateral hemisphere** | | | | |
| --- | --- | --- | --- | --- | --- | --- |
|  |  | **Speech perception** | | | **Speech comprehension** | |
|  |  | *R* | *P_FWE_* | *R* | | *P_FWE_* |
| **Left PT** | **Surface area** | 0.00 | >.99 | 0.07 | | >.99 |
|  | **Thickness** | -0.04 | >.99 | -0.07 | | 0.99 |
|  | **Myelin content** | -0.02 | >.99 | 0.03 | | >.99 |
|  | **NDI** | -0.08 | 0.62 | -0.05 | | >.99 |
|  | **ODI** | 0.02 | >.99 | 0.02 | | >.99 |
| **Right PT** | **Surface area** | 0.02 | >.99 | 0.01 | | >.99 |
|  | **Thickness** | 0.03 | >.99 | 0.02 | | >.99 |
|  | **Myelin content** | 0.02 | >.99 | -0.01 | | >.99 |
|  | **NDI** | 0.04 | >.99 | -0.01 | | >.99 |
|  | **ODI** | 0.02 | >.99 | -0.02 | | >.99 |

PT, planum temporale; HG, Heschl's gyrus; *P_FWE_*, corrected *P* value after Bonferroni correction; NDI, neurite density index; ODI, orientation dispersion index.

Supplementary file 1j. The difference in PT functional and structural metrices between groups with single and duplicated HG within each hemisphere (the effect size, Cohen’s D).

|  | **Left hemisphere** | **Right hemisphere** |
| --- | --- | --- |
| **Speech perception** | 0.20 | 0.04 |
| **Speech comprehension** | **0.37** | 0.12 |
| **Surface area** | **0.77** | **0.98** |
| **Thickness** | 0.02 | 0.17 |
| **Myelin content** | **0.57** | **0.65** |
| **NDI** | **0.30** | **0.67** |
| **ODI** | **0.41** | 0.14 |

NDI, neurite density index; ODI, orientation dispersion index.
